# Integrated analysis identities Rho GTPases related molecular map in patients with gastric carcinoma

**DOI:** 10.1038/s41598-023-48294-z

**Published:** 2023-12-05

**Authors:** Shaowei Ma, Ying Wang, Weibo Li, Shaofan Qiu, Xiangyu Zhang, Ren Niu, Fangchao Zhao, Yu Zheng

**Affiliations:** 1 Department of Gastrointestinal Surgery, The Second Hospital of Hebei Medical University, Shijiazhuang, 050000 China; 2 Department of Cardiology, Xingtai Third Hospital, Xingtai, 054000 China; 3 Department of Oncology, The Second Hospital of Hebei Medical University, Shijiazhuang, 050000 China; 4 Department of Thoracic Surgery, The Second Hospital of Hebei Medical University, Shijiazhuang, 050000 China

**Keywords:** Cancer, Immunology, Gastroenterology, Oncology

## Abstract

The intricate involvement of Rho GTPases in a multitude of human malignancies and their diverse array of biological functions has garnered substantial attention within the scientific community. However, their expression pattern and potential role in gastric cancer (GC) remain unclear. In this study, we successfully identified two distinct subtypes associated with Rho GTPase-related gene (RGG) through consensus clustering analysis, which exhibited significant disparities in overall survival and the tumor microenvironment. Subsequently, an extensively validated risk model termed RGGscore was meticulously constructed to prognosticate the outcomes of GC patients. This model was further assessed and validated using an external cohort. Notably, the high RGGscore group was indicative of a poorer prognosis. Univariate and multivariate Cox regression analyses unveiled the RGGscore as an autonomous prognostic indicator for GC patients. Subsequent external validation, utilizing two cohorts of patients who underwent immunotherapy, demonstrated a significant correlation between a low RGGscore and improved response to immunotherapy. Additionally, the expression levels of three genes associated with RGGscore were examined using qRT-PCR. Taken together, a pioneering RGGscore model has been successfully established, showcasing its potential efficacy in offering valuable therapeutic guidance for GC.

## Introduction

Gastric cancer (GC) holds the fifth position among the most prevalent malignancies globally and stands as the third leading cause of cancer-related mortality, predominantly attributable to its aggressive progression and propensity for distant metastasis^1^. Despite notable advancements in comprehensive therapeutic approaches, the formidable challenge of metastasis persists, posing significant obstacles to achieving favorable clinical outcomes^2^. In recent times, various therapeutic modalities, particularly immunotherapy, have emerged as indispensable constituents of cancer treatment, exhibiting remarkably potent efficacy in tumor defense^3^. Nevertheless, there is considerable heterogeneity in the response to drugs, even among patients exhibiting similar clinicopathological characteristics^4,5^, the current classification methods, especially the pathological TNM staging system, appear to be inadequate in accurately predicting the response to therapeutic. Therefore, there is an urgent need to develop a novel molecular signature that can accurately classify subgroups of GC patients who are more likely to derive therapeutic benefits from specific treatment regimens.

Rho GTPases play a crucial role in governing the architecture and kinetics of the cytoskeleton, thereby exerting control over cell adhesion, morphology, and the progression of the cell cycle^6^. Rho GTPases serve as pivotal molecular switches, transitioning between an inactived state bound to GDP and an activated state bound to GTP. This intricate regulatory process is orchestrated by the concerted involvement of guanine nucleotide exchange factors (GEFs), GTPase-activating proteins (GAPs), and GDP dissociation inhibitors (GDIs). GEFs play a crucial role in promoting the activation of Rho GTPase, while both GAPs and GDIs are instrumental in its inhibition^7^. Presently, the dysregulation of Rho GTPases has been associated with malignancy induction, cellular viability, invasion, and metastasis^8,9^. While the primary outcome of dysregulated Rho family signaling is generally recognized as pro-oncogenic effects, it is noteworthy that Rho family proteins may also participate in antitumorigenic processes. This intricate involvement of these proteins in cancer complicates their precise role within the context of malignancy^10,11^. Prior investigations have elucidated the prospective role of Rho family GTPases as therapeutic targets in cancer treatment. Nevertheless, a comprehensive investigation of Rho family GTPases in GC remains lacking. Therefore, in our current study, we aimed to systematically evaluate the expression patterns of Rho family GTPase-related molecules in publicly available databases, with the intent to ascertain their clinical significance and potential therapeutic implications for GC.

## Materials and methods

### Dataset and preprocessing

Three distinct and independent GC cohorts, obtained from the TCGA and GEO databases, were integrated into our analysis. To ensure the integrity and reliability of the data, we applied rigorous inclusion criteria, including: (1) primary GC cases, (2) availability of gene expression profiles and complete survival information, (3) absence of prior chemotherapy or radiotherapy treatment before surgery, and (4) minimum survival time exceeding 30 days. Ultimately, the analyzed cohorts consisted of 299 patients in the GSE66229 dataset, 182 patients in the GSE15459 dataset, and 339 patients in the TCGA dataset.

The gene expression data from the GEO database was annotated using the Affymetrix platform (GPL570). The ComBat algorithm, embedded in the “sva” package, was employed for batch effect removal in the GEO database. The newly formed queue was named the microarray cohort. For the TCGA-GC cohort, RNA-seq data in FPKM format was converted to TPM and annotated using the GENCODE database (version GRCh38). The cohort after sample screening was named RNA-seq cohort. The Rho GTPase-related genes (RGGs) were downloaded from the REACTOME_RHO_GTPASE_CYCLE entry in the MSigDB database, encompassing a total of 450 RGGs. Immunohistochemistry (IHC) data were exclusively sourced from the Human Protein Atlas (HPA) database. HPA database aggregates data from multiple studies, often without specifying the exact number of cases stained for each specific marker. Regarding the scoring of staining intensity, we relied on the HPA’s predefined categorization, which classifies staining intensity as negative, low, medium, or high.

### Acquisition and processing of the scRNA-seq data

The standardized GC scRNA-seq dataset GSE134520 was downloaded from the single-cell database TISCH. Quality control was performed using nFeature_RNA > 200, nFeature_RNA < 4000, and percent.MT < 5. After normalizing, the first 2000 height-variable genes of each sample were analyzed and the ScaleData function was employed for scaling. The RunPCA function was used to reduce the dimensions of principal component analysis (PCA). Then, the FindNeighbors and FindClusters functions were applied for cellular clustering and visual analysis. Each cell cluster was annotated with cellular lineage annotation based on the cellular lineage marker genes.

### Consensus clustering

Based on the expression profiles of prognostic RGGs (*p* < 0.05), an unsupervised consensus clustering analysis was conducted using the “ConsensusClusterPlus” software package. The optimal number of clusters was obtained according to the cumulative distribution curve and K-means. PCA confirmed the effectiveness of the clustering.

### Immunoinfiltration analysis and functional enrichment analysis

The algorithm of single-sample gene set enrichment analysis (ssGSEA) was employed to estimate the abundance of immune cells in various samples, thus reflecting the immunological microenvironment status and comparing the mRNA expression levels of immune checkpoint inhibitors (ICIs) among different groups. Differentially expressed genes (DEGs) among various subtypes were identified using the R package “Limma”, with the significance criterion set as |log2(FoldChange)|> 0.5 and adjusted *p* value < 0.05. After identifying the DEGs, enrichment analysis of Gene Ontology (GO) and the Kyoto Encyclopedia of Genes and Genomes (KEGG) was performed using the “clusterProfiler” package. A *P* value < 0.05 and q-value < 0.05 were selected as the criteria for discerning significantly enriched pathways.

### Construction and validation of the risk score model

The RNA-seq cohort was employed for modeling, while the microarray cohort was utilized for external validation. Within the RNA-seq cohort, univariate Cox regression analysis was employed to ascertain prognostic-related RGGs. The least absolute shrinkage and selection operator (LASSO) model was utilized to eliminate redundant genes. Correlation coefficients and gene expression values were obtained through multivariate Cox regression analysis, and a risk score calculation formula was established. According to the median score calculated using this formula, patients were divided into a high-risk group and a low-risk group. The prognostic value of risk scores in the RNA-seq and microarray cohorts was evaluated using univariate and multivariate Cox regression analysis. The R package “timeROC” was utilized to depict the receiver operating characteristic (ROC) curve and ascertain the area under the curve (AUC), thereby evaluating the prognostic effectiveness.

### Immunotherapy dataset

Using two cohorts treated with immune checkpoint blockade (ICB) therapies, namely IMvigor210 and GSE78220, to validate the use of a risk model for predicting the efficacy of immunotherapy. The IMvigor210 dataset comprised 298 individuals afflicted with advanced urothelial carcinoma who underwent treatment with the anti-PD-L1 medication, Atezolizumab^12^. In the GSE78220 dataset, patients with metastatic melanoma underwent treatment with the anti-PD-1 drug, pembrolizumab^13^.

### Cell culture and qRT-PCR analysis

The GC cell lines (HGC-27, SGC-7901, and BGC-823), as well as the human normal gastric mucosal epithelial cell line (GSE-1) were kindly provided by the Cell Repository of the Chinese Academy of Sciences (Shanghai, China). All cell lines were cultured in RPMI-1640 medium containing 10% Fetal Bovine Serum (FBS), streptomycin (100 U/mL), and penicillin (100 U/mL) at 37 °C in 5% CO_2_ atmosphere.

TRIzol^®^ (1 mL) was used to isolate total RNA from cell lines, and complementary DNA (cDNA) was created using reverse transcriptase from avian medulloblastoma virus and random primers according to TAKARA’s instructions. SYBR Premix Ex Taq II (Takara, Shiga, Japan) was adopted to perform qRT-PCR. Data were analyzed using 2^−ΔΔCT^ values. The primer sequences of GADPH were F: 5′-ACAGTCAGCCGCATCTTCTT-3′ and R: 5′-AAATGAGCCCCAGCCTTCTC-3′. The primer sequences of NCKAP1 were F: 5′-TTGTACCCCATAGCAAGTCTCT-3′ and R: 5′-GGGCATTTCTCCACTGGTCAG-3′. The primer sequences of PIK3R3 were F: 5′-TACAATACGGTGTGGAGTATGGA-3′ and R: 5′-TCATTGGCTTAGGTGGCTTTG-3′. The primer sequences of CPNE8 were F: 5′-ACATGACTTTCTGGGACAAGTG-3′ and R: 5′-GCATCCCTGCAACAGTTTAATTC-3′.

### Statistical analysis

The statistical analyses were executed using R software (version 4.0.1), as described earlier in this study. The significance level was set at a *P*-value below 0.05, indicating statistical significance.

## Results

### RGGs exhibit differential expression and prognostic predictive capacity in GC

**Figure 1:**
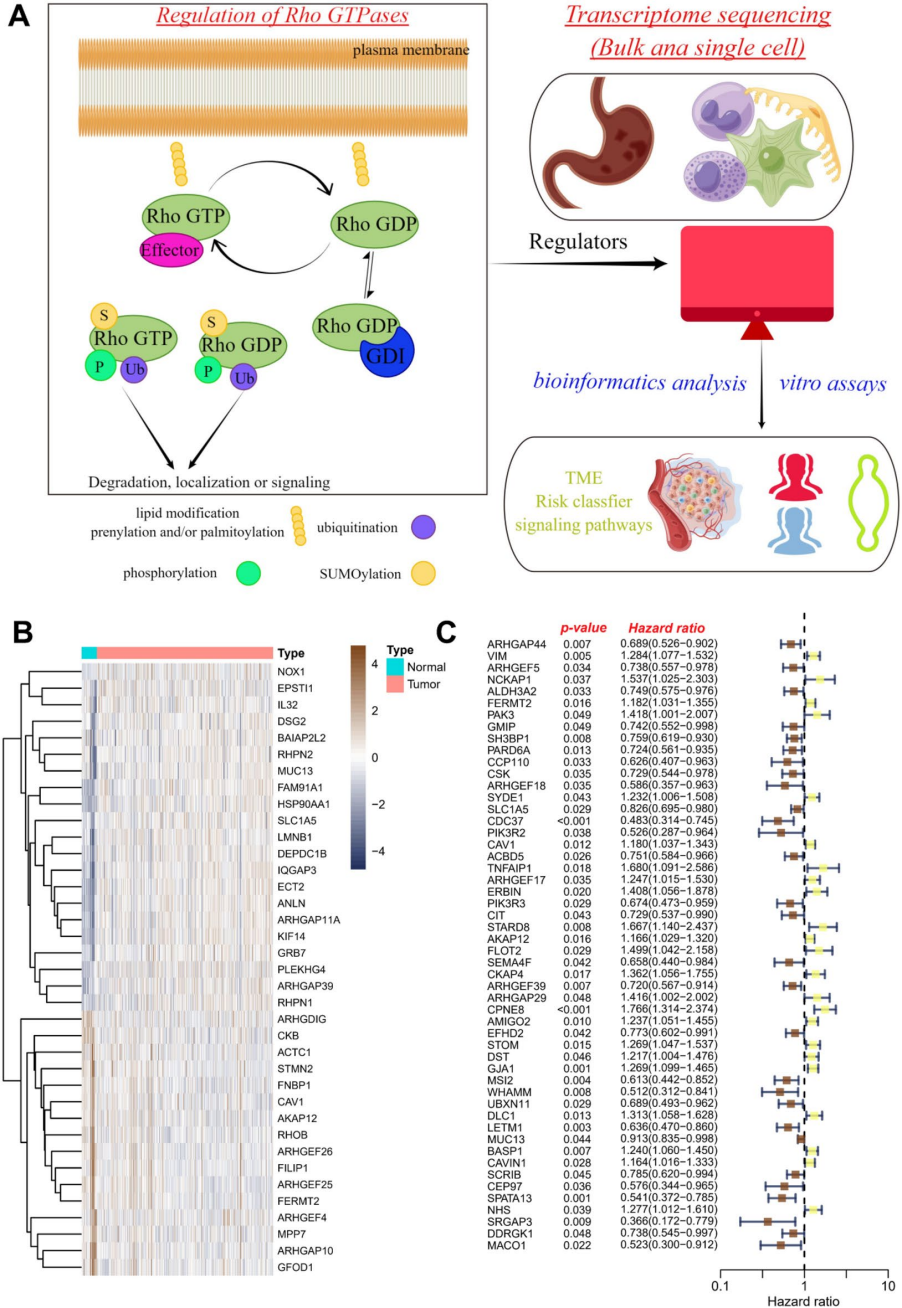
The identification of the candidate RGGs in the RNA-seq cohort. (**A**) Regulation of Rho GTPases and graphical abstract. (**B**) The heatmap of 37 differentially expressed RGGs in GC tissues. (**C**) Univariate Cox regression of RGGs for the screening of prognosis-related genes.

As depicted in Fig. 1A, a notable proportion of Rho GTPases are subject to regulatory control by GEFs, GAPs, and GDIs. Through their influence, these proteins govern the intricate cycling between the active GTP-bound conformation and the inactive GDP-bound conformation of Rho GTPases. Additionally, Rho GTPases were also regulated by post-translational modifications, including lipid modification, phosphorylation, ubiquitination, and SUMOylation, which profoundly impact their intracellular localization, stability, and ability to engage downstream effectors.

Firstly, we identified the differential expression of RGGs in the RNA-seq cohort. The findings revealed a total of 37 differential RGGs. Among them, 21 RGGs were up-regulated in tumor tissues and 16 RGGs were down-regulated in normal tissues. Notably, MUC13 up-regulated the most, while CKB down-regulated the most (Fig. 1B). Subsequently, in the RNA-seq cohort, univariate Cox regression analysis showed that a total of 52 RGGs had prognostic indicators, of which CPNE8 was the largest risk factor for HR and SRGAP3 was the strongest protective factor (Fig. 1C).

### Two distinct situations of Rho GTPase modification in the GC

**Figure 2:**
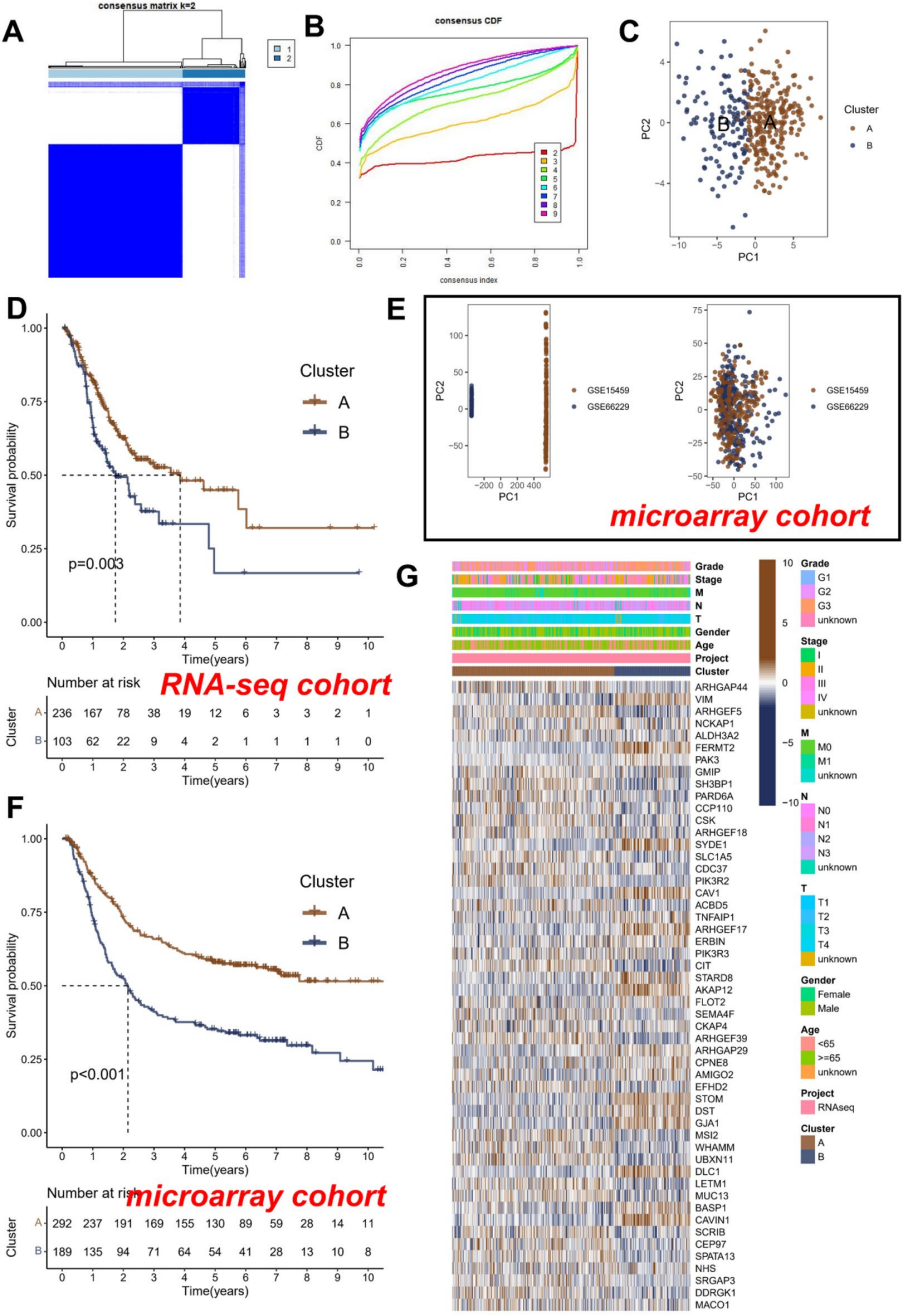
Consensus clustering analysis of 52 RGGs. (**A**) Heatmap of the consensus matrices for k = 2. (**B**) The CDF curves for clusters at k = 2 to 9. (**C**) PCA plot for the two clusters in the RNA-seq cohort. Kaplan–Meier curves based on two clusters in the RNA-seq cohort (**D**) and microarray cohort (**F**). (**E**) PCA analysis in the microarray cohort. (**G**) Heatmap and the clinical parameters of the two clusters.

Firstly, the expression profile comprised of the aforementioned 52 RGGs in the RNA-seq cohort was utilized, and all tumor samples were partitioned into k subtypes using the R software package “ConsensusClusterPlus”. According to the consensus score of the heat map (Fig. 2A) and the CDF curve (Fig. 2B), it could be inferred that k = 2 was the optimal grouping. Furthermore, PCA revealed pronounced transcriptomic heterogeneity in the aforementioned two subtypes (Fig. 2C), suggesting the potential existence of two distinct Rho GTPases modification scenarios within the context of GC. Survival analysis showed that subtype B had the worst prognosis (n = 103), while subtype A had the best prognosis (n = 236) (Fig. 2D).

To further validate the robustness of this classification, verification was conducted using a microarray cohort. The results demonstrated that the microarray cohort could be classified into two subtypes based on the expression profile derived from the same 52 RGGs (Fig. 2E). Among them, subtype A contains 292 patients, subtype B contains 189 patients, and also subtype B has the worst prognosis (Fig. 2F).

Finally, we demonstrated the correlation between the two subtypes and RGGs and clinical information in the RNA-seq cohort. The results showed that the possible reason for the two different Rho GTPases modification may potentially stem from differential expression of RGGs, such as subtype B exhibiting elevated expression of VIM, FERMT2, and other genes, whereas subtype A displayed heightened expression of ARHGAP44 and ARHGEF5 (Fig. 2G).

### Immune microenvironment and pathway differences of different Rho GTPases modification conditions

**Figure 3:**
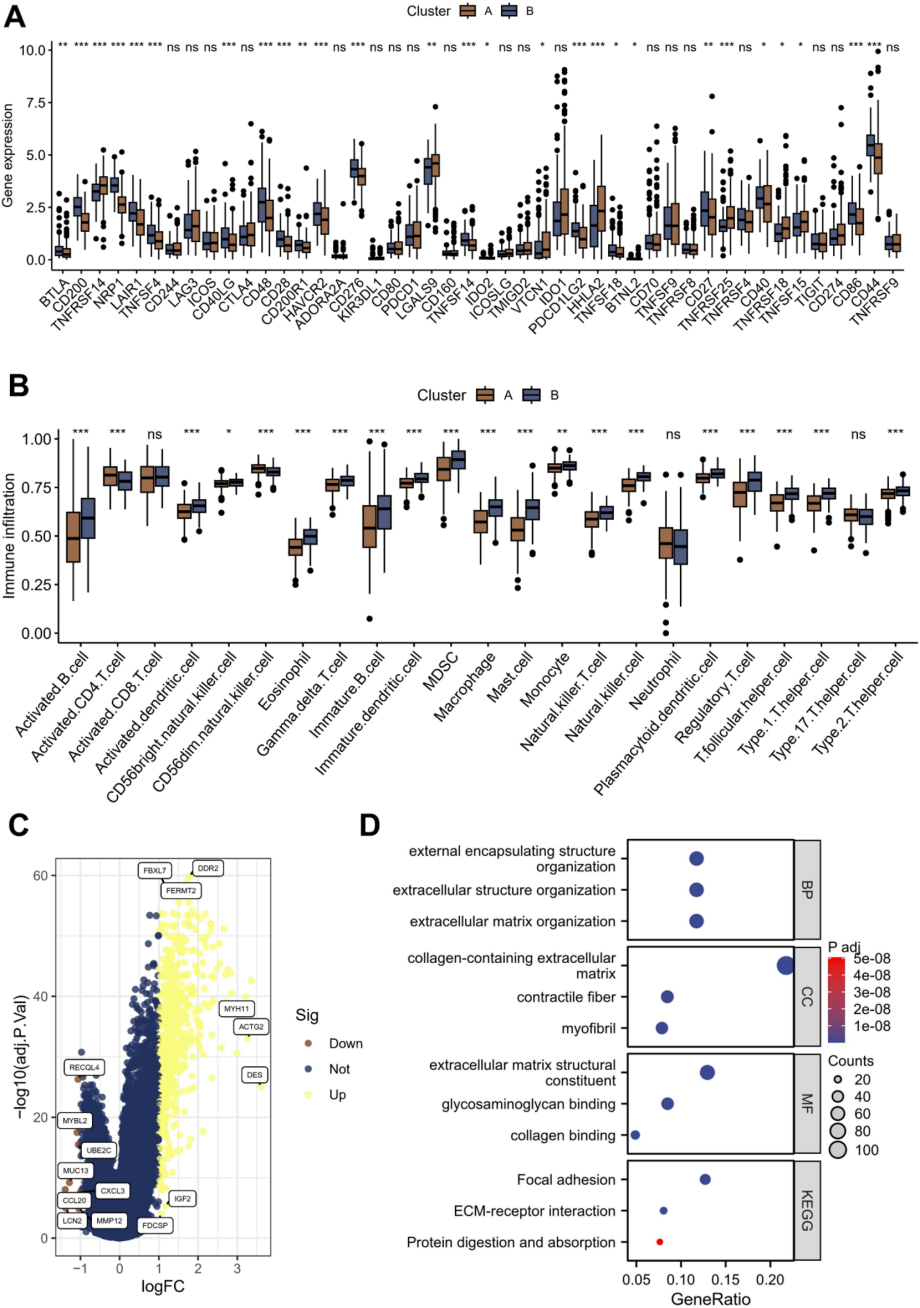
Analysis of immune microenvironment and pathway enrichment. (**A**) Differences in the abundance of immune-checkpoint-related genes between two subtypes. (**B**) Different immune cell infiltrations were compared between two subtypes. (**C**) Volcano plot showing DEGs between two subtypes. (**D**) GO and KEGG enrichment analyses of DEGs between two subtypes. ns not significant, *p < 0.05, **p < 0.01, ***p < 0.001.

We compared the expression patterns of ICIs in different subtypes. Interestingly, subtype B demonstrated higher mRNA expression in most ICIs, such as CD276 and HAVCR2. Conversely, certain ICIs showed elevated expression in subtype A, including TNFRSF14 and LGALS9 (Fig. 3A). Furthermore, we further refined the content of various immune cells in different samples by ssGSEA analysis. The box plot showed the difference in the content of immune cells in different subtypes. We observed a significant increase in most immune killer cells in subtype B. However, MDSC and Treg cells were also significantly up-regulated, which may cause an immunosuppressive environment in the tumor and provide a corresponding explanation for its worst prognosis (Fig. 3B).

To explore the reasons for the different survival status and immune landscape caused by different Rho GTPases modification, we analyzed the differences between the two subtypes. A total of 524 differentially expressed genes were identified, most of which were significantly up-regulated in subtype B, and only 8 genes were up-regulated in subtype A, namely RECQL4, MYBL2, UBE2C, MUC13, CCL20, CXCL3, LCN2 and MMP12 (Fig. 3C). Subsequently, we conducted KEGG and GO enrichment analyses on 524 differentially expressed genes. The results showed that most of them were concentrated in the extracellular matrix and were highly correlated with focal adhesion, ECM-receptor interaction and other pathways (Fig. 3D).

### Develop a Rho GTPases-related risk identification system for easy clinical use

Although two different Rho GTPases modifications were identified by the transcription profile of RGGs, and the heterogeneity of survival and biological functions was reflected. However, molecular subtyping was based on studies conducted on patient populations, thus rendering it incapable of accurately predicting the individual condition of each patient. Therefore, we evaluated the risk score for individual patients based on the expression profiles composed of RGGs in single-factor Cox regression analysis, aiming to facilitate clinical application.

**Figure 4:**
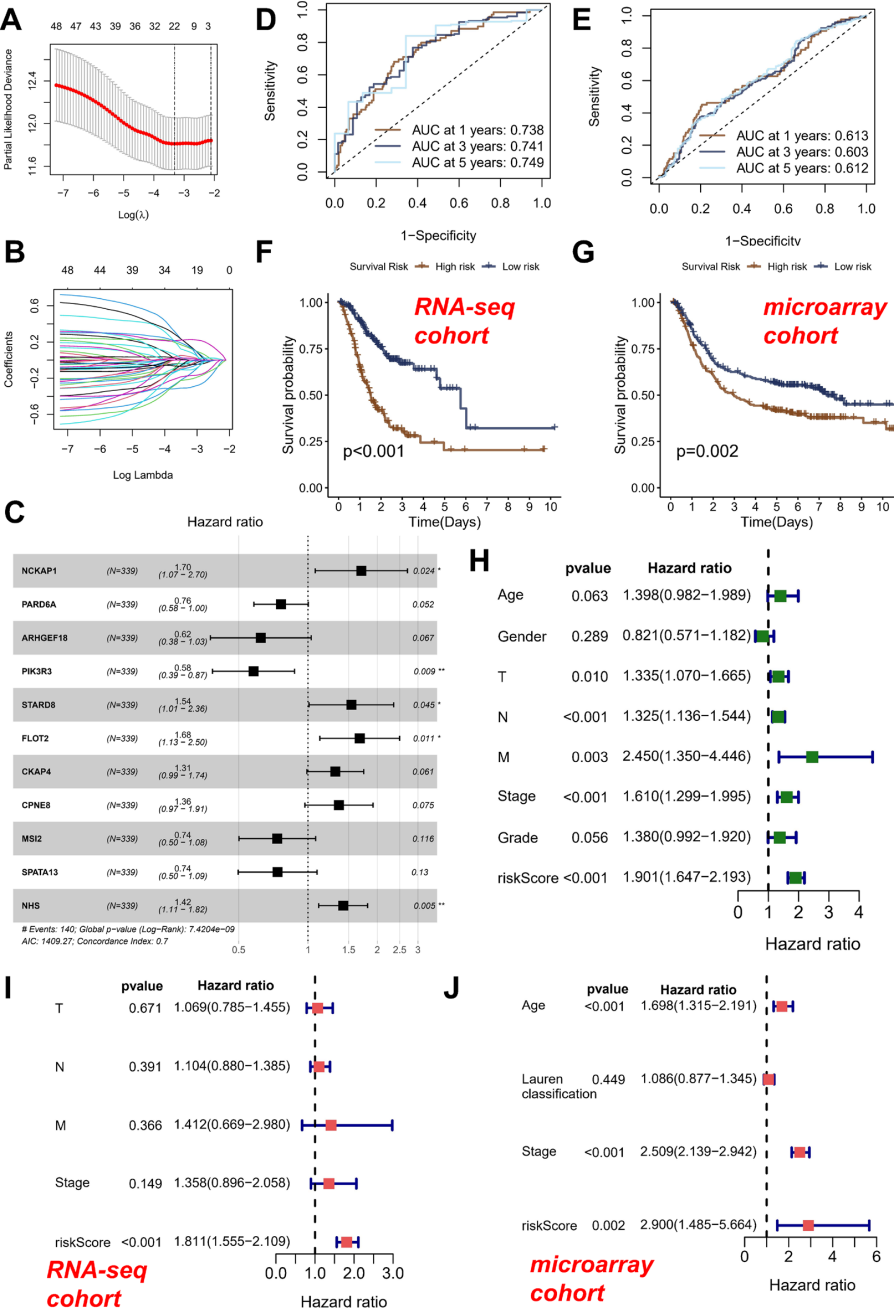
Development and validation of Rho GTPases related risk identification system. (**A**, **B**) LASSO Cox regression analysis to develop the prognostic model. (**C**) Forest plot of the 11 target genes that compose the RGGscore model. Time-dependent ROC of the RNA-seq cohort (**D**) and microarray cohort (**E**). Kaplan–Meier curves of the RNA-seq cohort (**F**) and microarray cohort (**G**). (**H**) Univariate Cox regression analysis of RGGscore in the RNA-seq cohort. Multivariate Cox regression analysis of RGGscore in the RNA-seq cohort (**I**) and microarray cohort (**J**). *p < 0.05, **p < 0.01.

First, utilizing LASSO regression analysis in the RNA-seq cohort (Fig. 4A,B), selectively eliminated redundant RGGs, and ultimately incorporated 21 RGGs into a multifactor Cox regression equation. Using the Akaike Information Criterion (AIC) to identify models that provided the optimal data explanation while containing the least free parameters. Ultimately, 11 genes were finally screened out and used to construct a risk model named RGGscore (Fig. 4C).

According to the median, it was divided into high-risk and low-risk groups. Table 1 displayed the coefficients within the equation. To verify the accuracy of RGGscore, we computed the risk score for each patient in the microarray cohort using the identical equation parameters. It was interesting to note that the ROC curves demonstrated the accuracy (ACU > 0.6) of survival prediction for 1, 3, and 5 years in both the RNA-seq cohort (Fig. 4D) and the microarray cohort (Fig. 4E). Similarly, survival analysis revealed that in the RNA-seq cohort (Fig. 4F) and microarray cohort (Fig. 4G), the prognosis of the high RGGscore group was inferior to that of the low RGGscore group.

**Table 1:** Results of prognosis related genes in RGGs in multivariate Cox regression.

id	coef	HR	HR.95L	HR.95H	*p* value
NCKAP1	0.5330	1.7040	1.0743	2.7027	0.0235
PARD6A	− 0.2693	0.7639	0.5822	1.0024	0.0520
ARHGEF18	− 0.4716	0.6240	0.3770	1.0329	0.0666
PIK3R3	− 0.5442	0.5803	0.3850	0.8747	0.0093
STARD8	0.4334	1.5425	1.0103	2.3551	0.0447
FLOT2	0.5168	1.6766	1.1256	2.4974	0.0110
CKAP4	0.2722	1.3128	0.9878	1.7447	0.0607
CPNE8	0.3093	1.3624	0.9696	1.9144	0.0747
MSI2	− 0.3074	0.7354	0.5014	1.0786	0.1157
SPATA13	− 0.3043	0.7377	0.4973	1.0943	0.1305
NHS	0.3518	1.4216	1.1134	1.8150	0.0048

To assess its prognostic predictive capacity, we conducted univariate and multivariate Cox regression analyses by incorporating additional clinical factors into the RNA-seq cohort (Fig. 4H,I). The results demonstrated that RGGscore possessed autonomous prognostic indicatory capacity and, moreover, exhibited the highest HR value of 1.811 among the factors under regression analysis. Similarly, within the microarray cohort, a multifactorial Cox regression analysis revealed that RGGscore (HR = 2.9) possessed the most robust capacity for independent prognostic indication (Fig. 4J).

### The risk identification system associated with Rho GTPases can provide guidance for immunotherapy

The emergence of immunotherapy, typically represented by PD-1/PD-L1 checkpoint inhibitors, signified a significant milestone in the treatment of tumors. Considering the potential therapeutic effects of immunotherapy, particularly PD-1 and PD-L1 immune checkpoint inhibitors, in various malignancies, including GC, we further assessed the predictive role of RGGscore in the IMvigor210 and GSE78220 cohorts.

**Figure 5:**
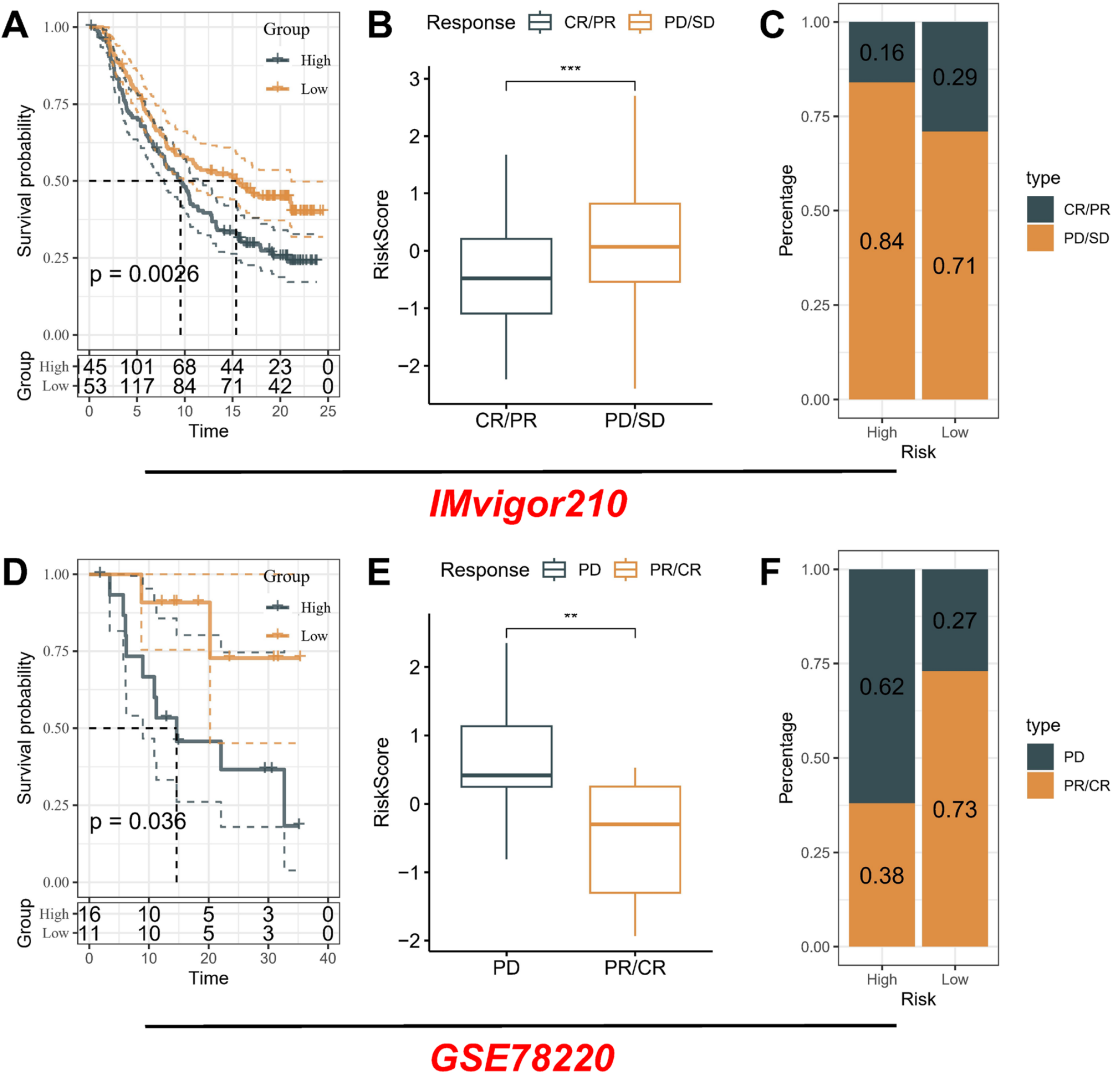
Correlation of RGGscore with immunotherapy response in two cohorts. Kaplan–Meier curves for patients with high and low RGGscore groups in the IMvigor210 cohort (**A**) and GSE78220 cohort (**D**). Difference in RGGscore among two immunotherapy response groups in the IMvigor210 cohort (**B**) and GSE78220 cohort (**E**). The distribution of immunotherapy response in indicated groups stratified by RGGscore in the IMvigor210 cohort (**C**) and GSE78220 cohort (**F**). **p < 0.01, ***p < 0.001.

On one hand, we assessed the correlation between RGGscore and immune therapy response in the IMvigor210 cohort, consisting of patients with advanced urothelial carcinoma undergoing PD-L1 blockade treatment. Compared to patients in the high RGGscore group, patients in the low RGGscore group exhibited evident survival advantages (Fig. 5A). The RGGscore of patients in the progressive disease (PD)/stable disease (SD) group were significantly higher compared to the partial response (PR)/complete response (CR) group (Fig. 5B). It is worth noting that the proportion of PR/CR in the high RGGscore group was significantly lower than that in the low RGGscore group. However, the proportion of PD/SD patients demonstrated an opposing trend, indicating that RGGscore could unveil patients' response to ICB therapy (Fig. 5C).

On the other hand, we conducted a similar analysis within the GSE78220 cohort comprising patients with melanoma undergoing PD-1 blockade therapy. Similarly, in comparison to patients in the high RGGscore group, those in the low RGGscore group exhibited conspicuous survival advantages (Fig. 5D). The RGGscore of the PD group was significantly higher compared to the PR/CR group (Fig. 5E). It was noteworthy that the low RGGscore constituted the predominant subtype (73%) within the PR/CR group, whereas the high RGGscore was a significant subtype (62%) within the PD group. This indicated that RGGscore held promise as a hopeful predictive indicator for the immunotherapeutic response in GC patients (Fig. 5F). Moreover, in TCGA cohort’s results, we found that not only the expression of PD-L1, but also among other immune checkpoints that were statistically significant, mRNA was higher in the low-RGGscore group (Supplementary Fig. 1), which may represent a better therapeutic response.

### Characterization of the single-cell level of the Rho GTPases-associated risk recognition system

**Figure 6:**
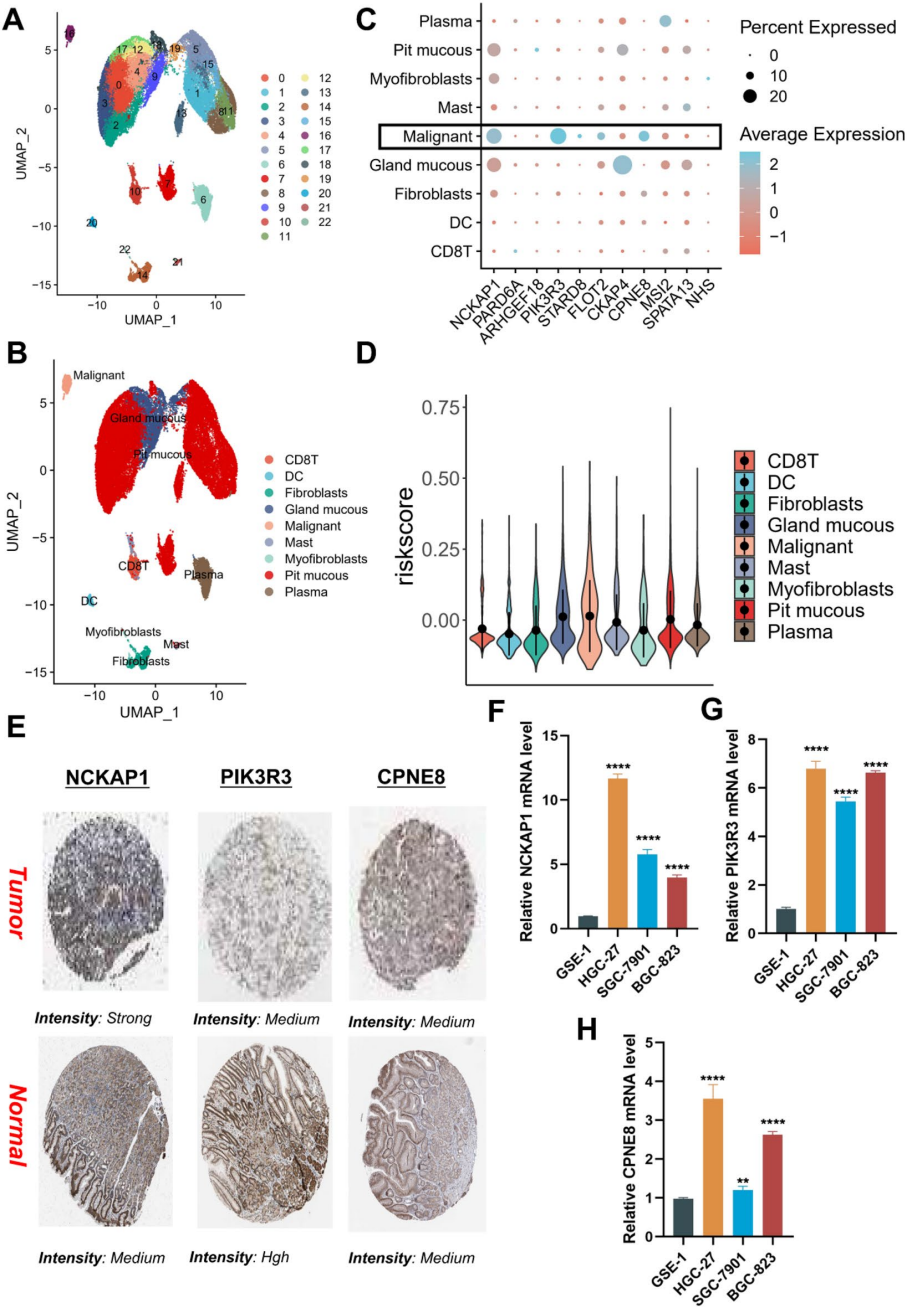
Validation of the expression of the signature genes in GC. (**A**) The results of the dimension reduction cluster analysis are shown in the UMAP diagram. (**B**) Cells were annotated into 9 different types of cells. (**C**) Expression of different risk genes in different cells. (**D**) The expression of Risk score in different cells. (**E**) The protein expression of the three genes in GC tumor tissues and normal tissues. (**F–H**) Further verification of the mRNA expression levels of three signature genes in human GC cancer cell lines and human normal gastric epithelial cell line by qRT-PCR analysis. **p < 0.01, ****p < 0.0001.

In order to further characterize the expression patterns of Rho GTPase-associated risk factors in different cells, we conducted an exploration using the GC single-cell data from the GSE134520 dataset. The findings revealed that the cells in this dataset could be classified into 23 subgroups (Fig. 6A). Using the annotation files from the TISCH database, the 23 subgroups were further divided into 9 cellular clusters, including CD8 T cells, dendritic cells, fibroblasts, glandular mucous cells, malignant cells, mast cells, myofibroblasts, pit mucous cells, and plasma cells (Fig. 6B).

Subsequently, we examined the expression of genes involved in the risk recognition system of Rho-GTPases, and the findings unveiled significant expression of NCKAP1, PIK3R3, and CPNE8 in malignant cells (Fig. 6C). Similarly, we conducted RGGscore for various cells, and the outcomes revealed that cells with high RGGscore exhibited characteristics of malignancy and glandular mucus, aligning with our suppositions (Fig. 6D).

Furthermore, based on the IHC data from the HPA database, we further examined the chromosomal localization of NCKAP1, PIK3R3, and CPNE8, revealing their pronounced staining within the glandular regions. This finding served to further substantiate our conclusion (Fig. 6E). Finally, based on cellular lineage validation, it was confirmed that NCKAP1, PIK3R3, and CPNE8 exhibited markedly elevated expression in GC cell lines compared to normal gastric mucosal epithelial cells (Fig. 6F-H).

## Discussion

Members of the Rho family of GTPases assume pivotal roles in a diverse range of cellular processes, including but not limited to cell proliferation, motility, cytoskeletal regulation, establishment of cellular polarity, and transcriptional regulation^14–16^. Several investigations have revealed the pivotal role of this gene family in both the initiation and progression of tumorigenesis as well as developmental processes^14^. They possess the capability to both facilitate tumor growth and inhibit tumor development^17^. To date, the relationship between overall survival, clinical characteristics, and the expression of Rho family GTPases in GC remains unexplored. Additional investigations are required to authenticate the exact function of these gene families in GC. This study aimed to conduct bioinformatics analyses in order to investigate the expression profile and prognostic significance of RGGs in GC, with the objective of improving the accuracy of prognosis prediction.

We employed a robust methodology to identify RGG subtypes using consensus clustering algorithms. This approach enabled us to capture a broad and representative spectrum of gene expression patterns associated with GC, thereby enhancing the relevance and applicability of our model. The findings of our study elucidate the significance of RGGs in GC, as we identified two distinct molecular subtypes based on the expression profiles of 52 RGGs. Notably, patients belonging to subtype A exhibited more favorable survival outcomes and clinicopathological characteristics. Furthermore, notable disparities in TME features were observed between the two subgroups. Subtype B exhibited a “hot” tumor phenotype characterized by heightened immune cell infiltration and elevated expression of ICIs mRNA, suggesting a potential enhanced responsiveness to immunotherapeutic interventions.

Utilizing DEGs, we constructed a robust prognostic model termed RGGscore, which is founded on a panel of RGGs. The RGGscore was meticulously calculated based on the expression levels of the identified genes. We ensured that this score was reflective of the overall survival disparities observed in GC patients. To confirm the reliability and accuracy of the RGGscore, we validated our model with external patient cohorts. This validation not only confirmed the reproducibility of our findings but also demonstrated the model’s effectiveness across different patient populations. This novel model aims to accurately predict the individualized prognosis of GC patients. The model consists of NCKAP1, PARD6A, ARHGEF18, PIK3R3, STARD8, FLOT2, CKAP4, CPNE8, MSI2, SPATA13, and NHS. NCKAP1 was first discovered in patients with Alzheimer’s disease (AD), which has been proved to drive metastasis in human non-small-cell lung cancer^18^. PARD6A has been established as an oncogene, and its association with multiple cancer types has been extensively validated^19^. ARHGEF18/p114RhoGEF is involved in tumorigenesis but the specific function has not been well investigated^20^. PIK3R3 has been shown to inhibit tumor cell senescence through p53/p21 signaling^21^. STARD8, a recently identified Rho GTPase-activating protein, exhibits downregulation in various malignancies and exerts inhibitory effects on tumor cell proliferation^22^. FLOT2 has been shown to assume a pivotal role in cancer cell proliferation, invasion, and migration, underscoring its significance in the progression of malignant diseases^23,24^. CKAP4 functions as a receptor for Dickkopf1 and participates in tumor progression^25^. CPNE8 has been implicated in the promotion of gastric cancer metastasis by modulating the focal adhesion pathway and the tumor microenvironment^26^. MSI2 plays a substantial role in tumorigenesis, rendering it a valuable prognostic marker and predictive indicator for chemotherapy response^27^. A previously conducted investigation has unveiled the involvement of Rho-family GEF Asef2 (SPATA13) in the modulation of cellular adhesion and actin dynamics, and thereby regulation of cell migration^28^. The function of NHS in non-small cell lung cancer has been identified as significantly associated with the processes of invasion and the development of liver metastasis^29^. As a result, the model incorporating these 11 genes exhibits promising potential for prognostic evaluation in GC. Robust evidence from both univariate and multivariate Cox regression analyses substantiated the RGGscore as an independent prognostic predictor, capable of providing valuable insights into the survival outcomes of GC patients. The ROC analysis further substantiated the robust predictive capacity of the RGGscore model for 1-, 3-, and 5-year OS in GC patients. Consequently, the RGGscore demonstrates considerable reliability as a prognostic indicator, offering valuable insights into the prognosis of GC patients.

Furthermore, accumulating evidence suggests that Rho GTPases contribute to the intricate crosstalk between tumor cells and their microenvironment. Through intricate interplay with various signaling molecules, Rho GTPases influence the tumor microenvironment, fostering an ecosystem that promotes cancer cell survival, immune evasion, and therapeutic resistance. This interplay underscores the intricate and dynamic nature of the tumor-stroma interaction, emphasizing the significance of Rho GTPases as potential therapeutic targets. The emergence of immunotherapy, typically represented by PD-1/PD-L1 checkpoint inhibitors, signified a significant milestone in the treatment of tumors. Considering the potential therapeutic effects of immunotherapy, particularly PD-1 and PD-L1 immune checkpoint inhibitors, in various malignancies, including GC, we further assessed the predictive role of RGGscore in the IMvigor210 and GSE78220 cohorts. Further validation was provided by correlating the RGGscore with the response to immunotherapy in two additional patient cohorts. The significant correlation observed underscored the potential of the RGGscore in guiding therapeutic decisions, thereby supporting its prognostic utility. On one hand, we assessed the correlation between RGGscore and immune therapy response in the IMvigor210 cohort, consisting of patients with advanced urothelial carcinoma undergoing PD-L1 blockade treatment. Compared to patients in the high RGGscore group, patients in the low RGGscore group exhibited evident survival advantages. The RGGscore of patients in the PD/SD group were significantly higher compared to the PR/CR group. It is worth noting that the proportion of PR/CR in the high RGGscore group was significantly lower than that in the low RGGscore group. However, the proportion of PD/SD patients demonstrated an opposing trend, indicating that RGGscore could unveil patients' response to ICB therapy. On the other hand, we conducted a similar analysis within the GSE78220 cohort comprising patients with melanoma undergoing PD-1 blockade therapy. Similarly, in comparison to patients in the high RGGscore group, those in the low RGGscore group exhibited conspicuous survival advantages. The RGGscore of the PD group was significantly higher compared to the PR/CR group. It was noteworthy that the low RGGscore constituted the predominant subtype (73%) within the PR/CR group, whereas the high RGGscore was a significant subtype (62%) within the PD/ group. This indicated that RGGscore held promise as a hopeful predictive indicator for the immunotherapeutic response in GC patients.

An additional noteworthy aspect of this study involves the in-depth exploration of the potential role of RGGs through the comprehensive analysis of single-cell data. This approach enables a finer-grained examination of the cellular heterogeneity and molecular dynamics underlying the influence of RGGs in the context of the studied disease. Through single cell clustering, the findings unveiled significant expression of NCKAP1, PIK3R3, and CPNE8 in malignant cells. Similarly, we conducted RGGscore for various cells, and the outcomes revealed that cells with high RGGscore exhibited characteristics of malignancy and glandular mucus, aligning with our suppositions. We validated the expression levels of the genes used in the model using qRT-PCR to ensure that the RGGscore was based on accurately measured gene expression data and that expression changes had occurred during tumorigenesis. Similarly, qRT-PCR was used to verify that the expression of NCKAP1, PIK3R3, and CPNE8 in GC cell lines was significantly higher than that in normal gastric mucosal epithelial cells. It is gratifying that the IHC data in the HPA database further prove our conclusion.

However, it is important to acknowledge the limitations of our research. Firstly, our developed model comprises a set of 11 genes, which may impose certain constraints on its wider clinical applicability. The current AUC value of our model, while promising, does not yet reach an optimal level. Therefore, further comprehensive analyses of RGGs are warranted to enhance the overall prognostic accuracy of our model. Future investigations should delve deeper into the intricacies of RGGs, aiming to refine and improve its predictive capabilities. Further investigations are also warranted to elucidate the molecular mechanisms underlying the impact of these signature genes on diverse prognostic indicators in patients with GC. Moreover, the data we collected and analyzed for RGGscore development were solely transcriptomic. We did not have access to corresponding IHC data that would allow us to directly measure protein expression levels, including PD-L1.

## Conclusion

In this study, we conducted a comprehensive and systematic analysis of RGGs, resulting in the development of a prognostic model referred to as RGGscore. This novel model was employed to investigate the prognostic implications, immune cell infiltration patterns, and potential immunotherapeutic responses in GC patients. Our findings offer valuable insights into the significance of RGGs and hold promise in the identification of novel therapeutic targets for the management of GC.

### Supplementary Information

Supplementary Legend.

Supplementary Figure S1.

## Data Availability

The original data for this study were obtained from the TCGA (https://portal.gdc.cancer.gov) and GEO (https://www.ncbi.nlm.nih.gov/geo/) databases. All data generated or analysed during this study are included in this published article and its supplementary information files. Further inquiries can be directed to the corresponding authors.
