# Supplementary material for: Integrated analysis identities Rho GTPases related molecular map in patients with gastric carcinoma
Source: Sci Rep. 2023 Dec 5;13:21443. doi: 10.1038/s41598-023-48294-z (PMC10698149; doi:10.1038/s41598-023-48294-z)
Supplement: Supplementary file 1 — Supplementary Legend. [file 41598_2023_48294_MOESM1_ESM.docx]

**Figure S1. Differences in immune checkpoint mRNA levels in different subgroups.****p* < 0.05, ***p* < 0.01, ****p*< 0.001.
