# Supplementary figures and images for: Integrated analysis identities Rho GTPases related molecular map in patients with gastric carcinoma
Source: Sci Rep. 2023 Dec 5;13:21443. doi: 10.1038/s41598-023-48294-z (PMC10698149; doi:10.1038/s41598-023-48294-z)

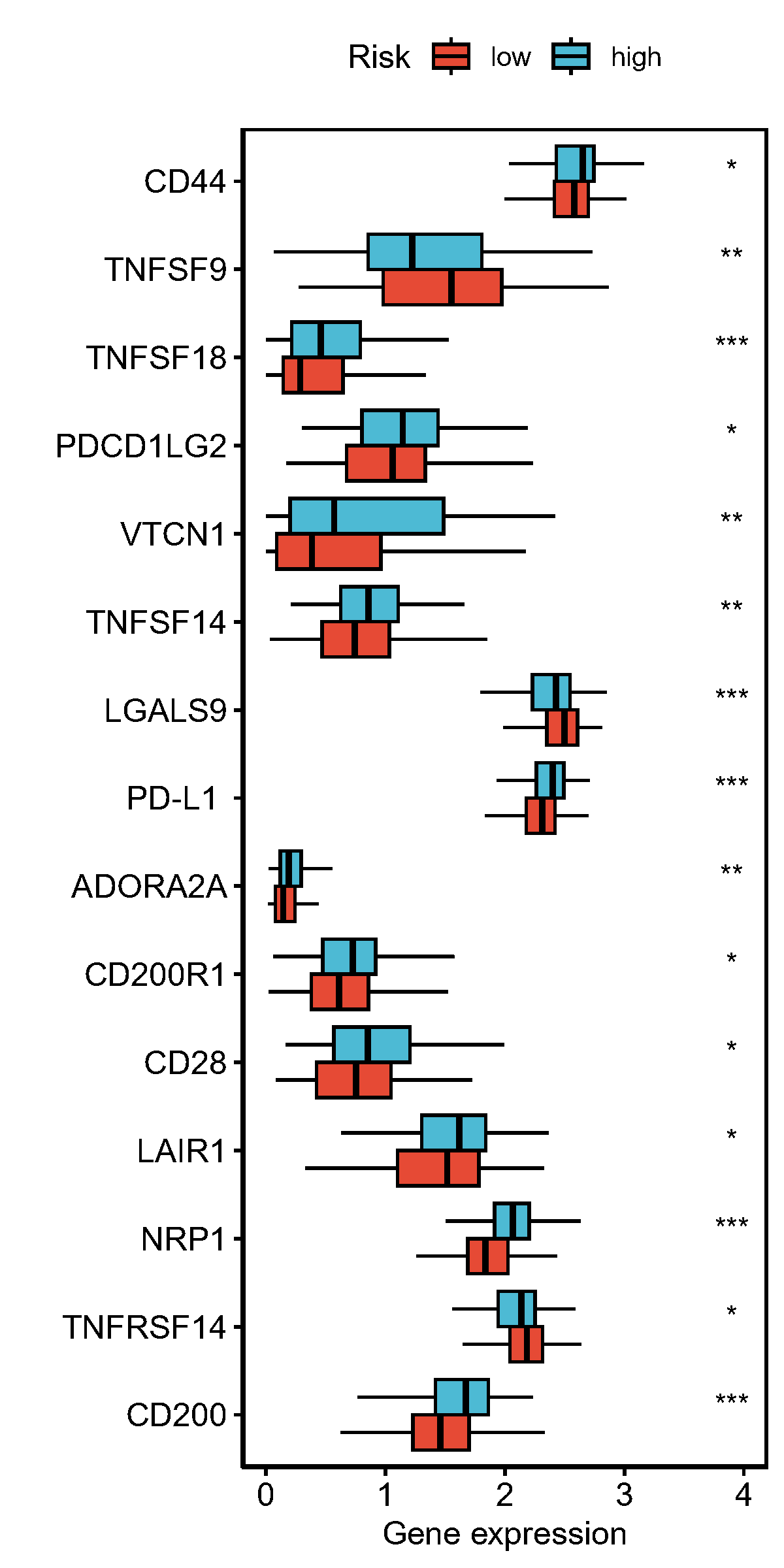

Supplement: Supplementary file 2 — Supplementary Figure S1. [file 41598_2023_48294_MOESM2_ESM.tif]
